# Copper-Doped Carbon Nanodots with Superior Photocatalysis, Directly Obtained from Chromium-Copper-Arsenic-Treated Wood Waste

**DOI:** 10.3390/polym15010136

**Published:** 2022-12-28

**Authors:** Dan Xing, Ahmed Koubaa, Yubo Tao, Sara Magdouli, Peng Li, Hassine Bouafif, Jingfa Zhang

**Affiliations:** 1 Forest Research Institute, Université du Québec en Abitibi-Témiscamingue, Rouyn-Noranda, QC J9X 5E4, Canada; 2 State Key Laboratory of Biobased Material and Green Papermaking, Qilu University of Technology, Shandong Academy of Sciences, Jinan 250353, China; 3 Centre Technologique des Résidus Industriels (CTRI), 433 Boulevard du Collège, Rouyn-Noranda, QC J9X 0E1, Canada

**Keywords:** carbon nanodots, CCA-treated wood waste, upcycling, sustainability, bioimaging, photocatalysis

## Abstract

An ecofriendly approach was developed for preparing copper-doped carbon dots (CDs) with superior photocatalysis using chromium-copper-arsenic (CCA)-treated wood waste as a precursor. Original wood (W-CDs), CCA-treated wood (C-CDs), and bioremediation CCA wood (Y-CDs) were used as the precursors. The chemical composition and structural, morphological, and optical properties, as well as the photocatalytic ability of the synthesized CDs varied with wood type. The C-CDs and W-CDs had similar characteristics: quasispherical in shape and with a diameter of 2 to 4.5 nm. However, the Y-CDs particles were irregular and stacked together, with a size of 1.5–3 nm. The presence of nitrogen prevented the formation of an aromatic structure for those CDs fabricated from bioremediation CCA wood. The three synthesized CDs showed a broad absorption peak at 260 nm and a weak absorption peak at 320 nm. Proof of the model study for the fabrication of luminescent CDs from CCA wood waste for bioimaging was provided. The degradation rate of CD photocatalytic MB was 97.8% for 30 min. Copper doping gives the CDs electron acceptor properties, improving their photocatalytic efficiency. This study provides novel ways to prepare nanomaterials from decommissioned wood as a nontoxic and low-cost alternative to fluorescent dots.

## 1. Introduction

Carbon nanomaterials, such as carbon nanodots (CDs) [1], carbon nanotubes [2], carbon nanofibers [3], and graphene [4], have attracted great attention in various fields. Among these nanomaterials, CDs in the shape of quasi spherical particles, with a size of around 10 nm, are mainly composed of carbon, hydrogen, and oxygen atoms [5]. CDs with the advantages of water solubility, low toxicity, photostability, cost-effectiveness, non-toxicity, eco-friendliness, biocompatibility, easy functionalization, and tunable photoluminescence have attracted widespread attention [6]. They are promising candidates compared to inorganic semiconductor quantum dots because of their advanced physicochemical properties [7]. CDs have been proven to have great potential for applications in biosensors, bioimaging, drug delivery, photocatalytic degradation, etc. [8]. 

Nowadays, various synthesis approaches for CDs have been proposed, including acidic oxidation, electrochemistry, hydrothermal carbonization, discharge, ultrasonic synthesis, solvothermal synthesis, laser ablation, and microwave pyrolysis [9]. However, those methods have several limitations, such as complicated multistep operations, limited spectral efficiency, low quantum yield, expensive cost, and harsh reaction conditions [7]. When considering the economic viability and environmental acceptability, the hydrothermal carbonization approach has gained much attention for preparing CDs. The previous literature shows that copper-doped fluorescent carbon dots can be obtained from l-tryptophan and copper chloride by a hydrothermal method [10].

Carbon nanodots have often been used for the photocatalytic degradation of organic pollutants [11]. Like other metal nanomaterials, carbon dots have excellent photocatalytic effects, especially semiconductor-doped carbon dots [12,13].

For sustainable development, an increasing number of natural precursors are recommended for fabricating CDs because of their abundance, ecofriendly character, and low cost. The reported natural precursors include actinidia deliciosa [14], soybean straw and cattle manure [15], grass [16], glucose [17], orange peels [18], citrus limetta [7], fungus [19], sago waste [20], etc. Of note, the properties of obtained CDs vary with different precursor materials. Therefore, other approaches to preparing CDs from various natural resources are worth exploring.

Wood utility poles are widely used in North America and Europe. They account for about 49.2% of the total global utility poles. Most wood utility poles are made from CCA-treated wood. CCA preservatives were initially designed to increase the resistance of the wood against microorganism decay in outdoor applications. However, owing to the high concentrations of toxic contaminants present in the CCA-treated wood and the increasingly large volumes of treated wood waste, it is urgent and important to find ways for the disposal of waste CCA wood [21]. The CCA-treated wood waste facilitates the uncontrolled release of these metals (loids) into soils and natural water [22]. It is important to recycle these wastes for environmental protection and sustainability.

Although many decontamination approaches have been investigated to reduce the adverse effect of CCA-treated wood, recycling still attracts increasing attention. The upcycling of solid waste is environmentally friendly, cost-effective, and low energy, which meets the requirements of sustainable development [23]. Various means have been used to reuse the waste of CCA-treated wood, such as reconstituted wood (e.g., particleboards, cement-bonded boards, and wood-plastic composites), recycling heavy metals, etc. [24]. Environmental protection and energy reuse are the main drivers of research into the decontamination and recycling of CCA-treated wood. However, there are few reports on upcycling CCA-treated wood waste at a high added value.

CCA-treated wood waste has been directly thermally transformed into biochar, which has good dielectric properties [25], and the properties of biochar conversed from CCA wood have also been investigated. Wood, consisting of cellulose, hemicellulose, and lignin, is a good carbon sink material. The author believes it will be a great precursor material for CDs. Compared to previous precursors (such as citric acid, ethylene glycol, and graphite), wood is a renewable natural product rather than a chemical. Using CCA-treated wood waste as a precursor to prepare carbon dots can reduce the utilization of petrochemical resources and help reduce carbon emissions.

This study explores obtaining CDs from CCA-treated wood via a hydrothermal carbonization method to explore their properties and utilization and compare them to that derived from the original wood. The synthesized CDs were characterized via high-resolution transmission electron microscopy (HR-TEM), X-ray photoelectron spectroscopy (XPS), X-ray diffraction (XRD), Raman spectroscopy, and Fourier transform infrared (FTIR) spectroscopy to explore their properties and potential application. The chemical structure and morphological changes of the synthesized CDs based on various precursor wood materials were studied. Meanwhile, *Saccharomyces cerevisiae* was studied to prove the nontoxic properties of the obtained CDs for further bioimaging applications. The novel copper-doped carbon dots directly obtained from CCA-treated wood waste were considered a nontoxic and low-cost alternative to nano-photocatalysts and fluorescence nanodots. The CCA-treated wood waste was upcycled to fabricate fluorescent C-CDs without using any other industrial chemicals or metals. This approach reduced the environmental hazards caused by solid wood pollutants while achieving the high-value reuse of waste wood.

## 2. Materials and Methods

### 2.1. Materials and Medium

This study used three kinds of wood samples (Pinus koraiensis Sieb. et Zucc.) to prepare CDs, including original wood, CCA-treated wood, and bioremediation CCA wood. The obtained CDs were respectively labeled as W-CDs, C-CDs, and Y-CDs. A commercial wood served as the control group. Tred’si (Westbury, QC, Canada) supplied the waste CCA-treated wood. The Global Bioresource Center provided the Yarrowia lipolytica 20460. Saccharomyces cerevisiae was procured from Angel Yeast, Yichang, China.

The mentioned bioremediation wood was the CCA wood treated with the Yarrowia lipolytica under the strain concentrations of OD = 0.6 for 15 days. In this case, the copper, chromium, and arsenic removal rate from the CCA-treated wood was 91.3%, 58.6%, and 32.8%, respectively. In brief, the strains were first cultivated in a yeast malt (YM) medium for 48 h. Then, the activated strains were transferred and cultivated in a growth medium in a shaker (200 mL medium/500 mL bottle). The YM medium was composed of yeast extract of 3 g, malt extract of 3 g, glucose anhydrous of 10 g, 5 g of tryptic soy broth, and 1 L of deionized water. The growth medium contained 50 g of glucose, 0.25 g of (NH4)_2_SO_4_, KH_2_PO_4_ of 1.7 g, NaH_2_PO_4_ of 12 g, MgSO_4_·7H_2_O of 1.25 g, yeast extract of 0.5 g, and deionized water of 1 L.

### 2.2. Carbon Nanodots Preparation

The three kinds of wood samples were first grounded into powders. Then, 2 g of each sample was placed in a 100 mL Teflon-lined autoclave. Afterward, deionized water of 80 mL was added to each autoclave. The mixture was agitated with a glass rod, and then the autoclave was heated using an oven at 200 °C for 6 h based on a previous report [26]. The CDs were obtained by removing the large particle biochar precipitation by centrifugation at 10,000 rpm for 15 min (Figure S1). A part of the synthesized carbon dots was dispersed in aqueous, and another was over-dried at 105 °C for further characterization and use. The pH value of the obtained CD solution was about 2.78 at 25 °C.

### 2.3. Cytotoxicity Evaluation

In this experiment, Saccharomyces cerevisiae was added into 250 mL flasks containing a YPD liquid medium of 100 mL to activate the strains. All flasks were incubated at 30 °C on a rotary shaker with a speed of 120 rpm for 24 h. An assay was performed to compare the morphology and the numbers of Saccharomyces cerevisiae cells on YPD agar plates supplemented with or without the synthesized C-CDs (as an example). Specifically, the As-prepared C-CDs solutions of different concentrations (0, 5, 10, 25, 50, and 75 μL·mL^−1^) were added to a solid YPD medium. Afterward, the activated strains were diluted 10,000 times, and 100 μL of the diluted strains was coated on the solid agar plates for cultivation for 24 h. The cellular toxicity experiment was performed in three replicates. The composition of the liquid YPD medium was mainly 1% yeast extract, 2% peptone, and 2% dextrose. The solid YPD medium was prepared by adding 2% agar to the liquid YPD medium. All mediums and materials were sterilized by high-pressure steam at 121 °C for 30 min before inoculation.

### 2.4. Photocatalysis Procedure

A mixture of 0.1 g TiO_2_ and 5 mL CDs was stirred for 4 h using a magnetic stirrer. Then, the mixture was prepared by over-drying at 100 °C to obtain the CDs/TiO_2_ composites. The catalysts of 30 mg were added into a glass beaker with the MB dyes of 30 mL with a concentration of 10 mg·L^−1^. The pH of the MB solution was 6.28. The mixture solution was magnetically stirred at room temperature under artificial visible light from a bulb or ultraviolet (UV) light. The power of the UV light used is 20 W, with a wavelength of 365 nm. The samples were collected after a set time and filtered through filter membranes of 0.22 μm. The absorbance value of the filtered supernatant was measured by a UV/vis spectrophotometer at 664 nm. All the experiments were performed in triplicate, and all results were marked as the mean ± standard deviation.

### 2.5. Measurement and Characterization of the Obtained CDs

HR-TEM (JEM 2100, JEOL, Tokyo, Japan) analyzed the micromorphology of the obtained CDs. Sample analysis was performed by depositing the synthesized CD droplets on a carbon-coated Cu grid. When the Cu grid was dried at an ambient temperature, the CDs were coated on the Cu grid. Images were obtained with a 200 kV voltage and a beam current of 105 μA. A fluorescence spectrophotometer (Cary Eclipse, Agilent Technologies, Santa Clara, CA, USA) was used to record the fluorescence spectra of the CDs. Spectra were acquired in a 1 cm quartz cuvette under the preset wavelength. The excitation wavelength varied from 260 to 400 nm. The excitation and emission slits were set to 5 nm with a voltage of 600 V. The Raman spectra were recorded with RENISHAW inVia spectrometer (Gloucester, UK) at 532 nm, with a laser intensity of 0.05% and a wavelength range from 400 to 3200 cm^−1^. The phase structure of the CDs was characterized using an X-ray diffractometer (Rigaku smartlab, Tokyo, Japan) with Cu Kα radiation (λ = 1.5418 A) at 40 kV and 30 mA. The scanning speed was set as 20° min^−1^, and the 2θ ranged from 5 to 80°. Element analysis of the CDs was carried out using XPS equipment (Thermo Scientific ESCALAB Xi, Waltham, MA, USA), and the Avantage software served to deconvolve the XPS spectra. XPS analyzed the As-prepared CD carbon, oxygen, nitrogen, copper, chromium, and arsenic elements. The fluorescence images were taken by a Leica fluorescence microscope (Weztlar DFC450C+DMI3000B, Weztlar, Germany), for which the samples were prepared by transferring the sole strain from the solid plate medium to a glass slide. CD Functional groups were recorded through FTIR, employing an IRTracer-100 with an ATR module (SHIMADZU Co., Ltd., Kyoto City, Japan). The analysis was performed with the spectra varying from 4000 to 500 cm^−1^ at a resolution of 4 cm^−1^ with an average of 64 scans.

## 3. Results and Discussion

### 3.1. Morphological and Structural Characterization of the CDs

**Figure 1:**
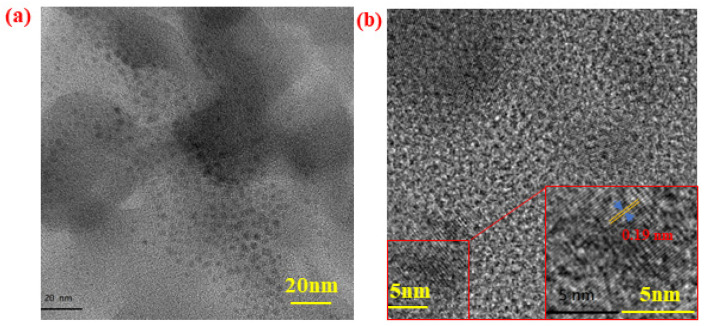
HR-TEM images of the synthesized C-CDs (**a**,**b**) and a zoomed-in image inside the red wireframe.

**Figure 2:**
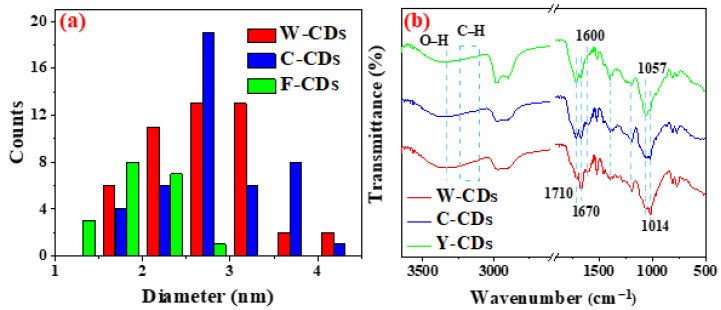
Size distribution of the As-prepared CDs (**a**) and the FTIR spectra of the CDs (**b**).

The HR-TEM images presented in Figure 1a,b and Figure S2a,b showed that the C-CDs and W-CDs appeared to have similar characteristics. The images revealed a homogeneous and well-dispersed population of carbon dots that are quasispherical and range from 2 to 4.5 nm in diameter. The sizes of the nanoparticles were estimated from the TEM results, averaging about 50 samples (Figure 1a and Figure 2a). The obtained sizes of the CDs were similar to those in previous reports [26]. The type of natural plant (precursor) has no obvious effect on the size and lattice fringes of the prepared CDs. Both the C-CDs and W-CDs exhibited lattice fringes with good resolution, spaced at 0.19 and 0.18 nm, respectively (Figure 1b and Figure S2b), which fit with the (100) crystallographic facet of graphitic carbon and indicates that both possess similar structures [27]. This result demonstrated no effects of the metals in the CCA-treated wood on the morphology of the synthesized carbon dots compared to the original wood. Interestingly, the Y-CDs particles were irregular in shape and were stacked together with a size of 1.5–3 nm (Figure 2a and Figure S2c,d). As expected, the fungi treatment influenced the forming process of the CDs. This can be explained by the fact that fungi treatment changed the morphology and structure of the CCA wood, as shown in Figure S3. The cells of *Y. lipolytica* contain lots of heteroatoms and adhere to the CCA wood surface, which caused a difference in the forming process of the Y-CDs. The presence of nitrogen changed the molecular structure of the CDs, which has been reported before [28]. The Y-CDs possessed well-resolved lattice fringes, spaced at 0.20, similar to those of the C-CDs and W-CDs. Commonly, the size and lattice fringes affect CD optical, morphological, and photocatalytic properties [29]. However, the doping of electron acceptors or e donors (different atoms or molecules) has a stronger effect on the CD properties than the size and lattice fringes [8].

The FTIR results indicated that various functional groups were present in the As-prepared CDs. As shown in Figure 2b, the W-CDs, C-CDs, and Y-CDs exhibited a broad absorption band at 3328 cm^–1^, attributed to the O–H stretching vibration mode. The antisymmetric and symmetric stretching vibration C–H bands appeared at 2976 and 2898 cm^−1^, respectively [30]. Previous studies have observed similar results [31,32]. Within the range of 1000–1100 cm^−1^, the spectrum of the W-CDs and C-CDs appeared to show a characteristic peak at 1014 cm^−1^ for C–O stretching vibrations, whereas the Y-CDs were blue-shifted to 1057 cm^−1^ due to the existence of N elements [14]. The W-CDs and C-CDs exhibited absorption bands at 1600 cm^−1^ corresponding to aromatic ring skeleton vibrational stretching, while the same absorption peak was not observed in the Y-CDs. It can be explained by the fact that the nitrogen doping prevented the formation of the aromatic ring skeleton in the Y-CDs. This can also explain the blue shift of the stretching vibration of carbon-oxygen bonds. The peaks appeared at 1670 and 1710 cm^−1^, corresponding to conjugated carbonyl and nonconjugated carbonyl stretching vibrations, respectively [33]. The peak intensity of the Y-CDs located at 1670 cm^−1^ was weaker than that of the W-CDs and C-CDs, whereas the peak at 1710 cm^−1^ is the opposite. It was ascribed to the low aromatic ring content in the Y-CDs, reducing the conjugation effects. This observation agreed with previous reports that precursor materials impacted the production of CD functional groups [26]. Peaks at 1190 and 1398 cm^−1^ corresponding to ether bonds and C–H bending were observed in the FTIR spectra, which is consistent with previous reports [34,35]. A previous study revealed that hydrophilic groups, such as –OH and –COOH, improve the solubility and stability of CDs [30]. The obtained CDs from the wood waste had various functional groups, including aromatic rings, hydroxyls, carboxylics, and carbonyl moieties, which benefit its biocompatibility. These CDs had more groups than those from graphite [36]. In addition to the size, the surface functional groups of carbon dots are also an important factor affecting their optical properties [29].

**Figure 3:**
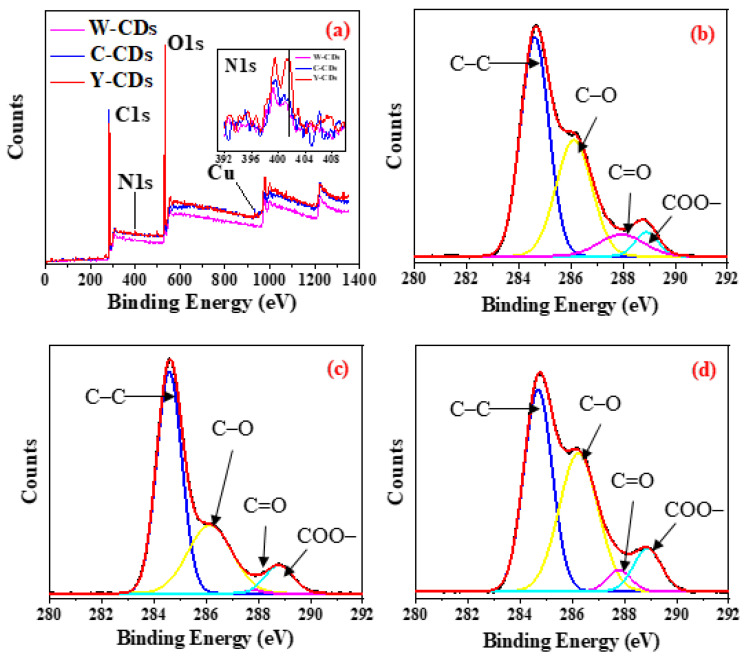
XPS full scan survey spectrum of the synthesized CDs and partially enlarged XPS spectrum of N1s in upper right illustration (**a**); high-resolution XPS spectrum of C1s for W-CDs (**b**), C-CDs (**c**), and Y-CDs (**d**).

As shown in Figure 3a, the full spectrum scan results revealed two main elements: C1s at 283 eV and O1s at 531 eV. Remarkably, a difference in the amount of Cu between the W-CDs, C-CDs, and Y-CDs was observed. This difference is because the CCA-treated wood precursor contains Cu ions. However, Cr and As were not observed in the obtained C-CDs, although they are also present in CCA wood waste because the minute quantity of Cr and As was difficult to detect. Cu was also not observed in the Y-CDs. The process of biological decontamination removed most of the heavy metal ions.

Another distinction was shown in the enlarged picture of N1s (Figure 3a), in which the peak of N1s at 399 eV only existed in the Y-CD spectrum, excluding the W-CDs and C-CDs. The SEM images (Figure S3) show that the surface of the CCA-treated wood had remaining yeast residues. This was the reason why the functional groups of the Y-CDs possessed N elements. This is in good agreement with the above FTIR results. In this way, a clean nitrogen-doped CD product was obtained by combining bioremediation and the hydrothermal conversion process for hazardous CCA wood waste. 

All samples displayed the presence of four peaks in the C1 bands at around 284.6, 286.2, 287.8, and 288.8 eV, which were ascribed to C–C, C–O, C=O, and O–C=O groups, respectively (Figure 3b–d). The peak intensity of the functional groups varied with CD type. The content of C–O and C=O for the C-CDs was lower than that of the other two nanomaterials. The C1s content of the C-CDs, W-CDs, and Y-CDs was 74.1%, 66.1%, and 63.0%, respectively. This result indicated that the CCA-treated wood was easier to transform into CDs than the original wood and bioremediation wood. This can be explained by the fact that heavy metals in the wood promoted the formation of CDs. The presence of the C1s peak at 284.6 eV suggested that the hydrothermal CDs originated predominantly from sp^2^ carbons [14]. The low oxygen content of the C-CDs was consistent with the high degree of carbonization during hydrothermal treatment (Figure S4).

**Figure 4:**
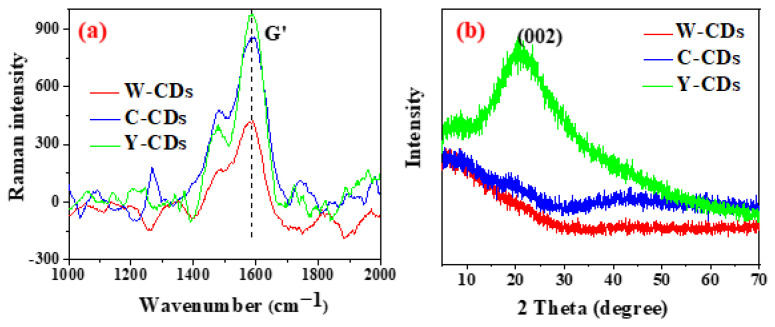
Raman spectrum (**a**) and XRD pattern of the synthesized CDs (**b**).

Raman spectroscopy is commonly used to characterize carbon materials to confirm the form of the carbon atom. As shown in Figure 4a, the Raman spectra of the W-CDs, C-CDs, and Y-CDs showed similar results, exhibiting a G-band. In general, D-bands are associated with the vibrational modes of carbon atoms in sp^3^ orbitals, and G-bands are associated with sp^2^ carbon atoms in a two-dimensional hexagonal lattice [26]. The presence of the G-band demonstrated that the main nature of the CDs constituted sp^2^ carbon atoms [18]. A possible reason for the absence of the D-band in the obtained CDs was that the sample’s states (for analysis) were different. The TEM sample was liquid, while the Raman sample was solid, obtained by drying the liquid sample. The aggregation of the nanomaterials changed the structure of the CDs, like the dehydration condensation of hydroxyl and carboxyl and the esterification reaction, resulting in the disappearance of the graphitic structure. Similar conclusions were drawn from the XRD measurements, which evaluated the crystallinity of the CDs. It is well known that crystal structure and grain size have a great influence on the optical and photocatalytic properties of nanomaterials [37,38]. As shown in Figure 4b, the XRD patterns of the Y-CDs showed an amorphous peak at 2θ = 20.7°, revealing a noncrystalline carbon phase in the CDs [39]. The broad reflection at around 21° could be ascribed to the graphitic carbon (002) plane. Those peaks disappeared in the spectra of the W-CDs and C-CDs. The difference was attributed to the different functional groups and elemental contents of the CDs, as discussed in the above XPS results. The appearance of the broad reflection for the Y-CDs could be attributed to the absence of aromatic rings, which was observed for the C-CDs (Figure 2b). All the above results indicated that it is feasible to prepare sustainable carbon nanomaterials from wood waste. 

### 3.2. Optical Properties

**Figure 5:**
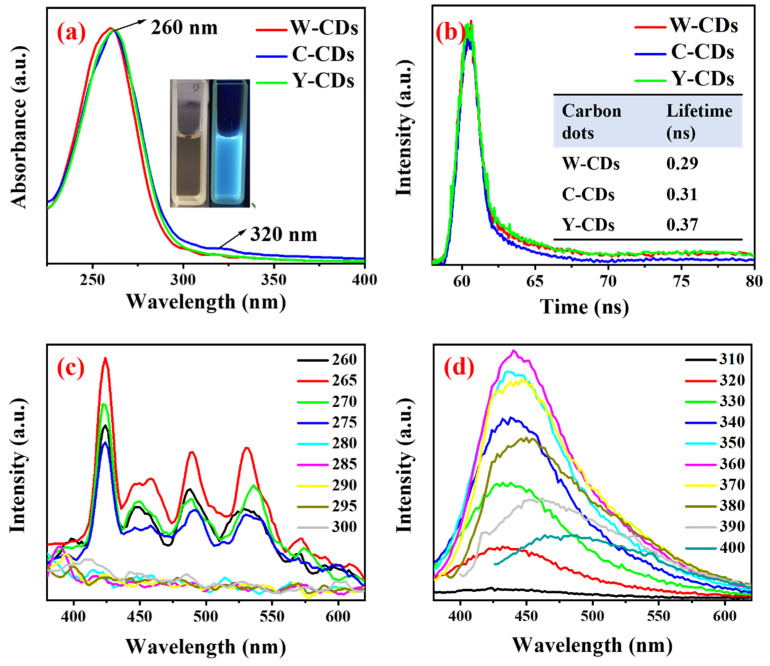
UV absorption spectra of the As-prepared CDs and fluorescence image of the C-CDs in visible light and at 365 nm UV light in upper right illustration (**a**); time-resolved photoluminescent measurements of the synthesized CDs (**b**); fluorescence spectra of the synthesized C-CDs at different excitation wavelengths from 260 to 300 nm (**c**) and from 310 to 400 nm (**d**).

The optical properties of the As-prepared CDs were analyzed in detail by ultraviolet (UV)-vis absorption and photoluminescence spectrometers. All the nanomaterials of the W-CDs, C-CDs, and Y-CDs revealed a broad absorption and a weak absorption peak in the UV band, respectively, located at 260 nm and 320 nm (Figure 5a). The presence of the peak at 260 nm could be attributed to the conjugated C=C with π − π* transition [40]. The peak at about 320 nm was ascribed to n→π* and π→π* molecular transitions for the C–C/C=O molecules in the CD architecture [39]. The UV-vis results further confirmed the formation of the CDs derived from the three biomass materials. The diluted CD solution was light brown under visible light (Figure 5a). In contrast, it turned bright blue under UV light at 365 nm.

The excitation spectra showed that the W-CDs, C-CDs, and Y-CDs had two peaks at 265 nm and 360 nm (Figure 5c,d and Figure S5). Specifically, for all the CDs, the emission intensity first increased and then decreased with increasing excitation wavelength (from 260 to 300 nm). The maximum emission intensity at 265 nm was observed when the excitation wavelength was 425 nm (Figure 5c and Figure S5a,c). However, for these three nanomaterials, the downward trend for intensity was different compared to the emission intensity at the same excitation wavelength of 260 nm. This is because these nanomaterials have various functional groups, molecular structures, and particle sizes.

The fluorescence intensity of the W-CDs, C-CDs, and Y-CDs increased first and then decreased with excitation wavelength, ranging from 310 nm to 400 nm. At the same time, the emission peaks were red shifted to longer wavelengths with increasing excitation wavelengths (Figure 5d and Figure S5b,d). The strongest emission intensity was noticed at an excitation wavelength of 360 nm, with an emission wavelength of 440 nm. The effect of excitation wavelength on the emission spectra of the CDs demonstrated their excitation-dependent emission behavior. This was ascribed to various reasons, including CD size, surface state distribution, oxygen-containing functional groups, and surface energy traps [32]. These observations were jointly influenced by emissive traps, triplet carbenes, exactions of carbon, aromatic conjugate interaction, and attached organic functional groups [32].

Time-resolved luminescence was used to assess the lifetimes of the synthesized CDs (Figure 5b). The luminescence decays of all the prepared CDs showed multiexponential behavior. The average lifetime (τ) is ordered as follows: Y-CDs > C-CDs > W-CDs, with values of 0.37, 0.31, and 0.29 ns, respectively. This observation showed that heteroatom (Cu and N) doping increased the fluorescence lifetime of the carbon dots. The reason is that the presence of heteroatom changes the surface chemical structure, reducing the exciton binding energy of the CDs compared with the previously reported CDs [27]. In the current study, the prepared CDs derived from CCA wood had a shorter luminescence lifetime than the CDs in the previous literature [28]. Nanocarbon dots with a short luminescence lifetime represent a great potential candidate for color converters in high modulation bandwidths for visible light communication [27]. This revealed a potential use for CDs obtained from CCA wood waste in visible light communication.

### 3.3. Cytotoxicity Studies, Bioimaging, and the Photocatalysis Applications of CDs

Due to their naked visible fluorescent properties, CDs in bioimaging have attracted more attention from researchers over the last few years. However, the cytotoxicity of CDs was a nonnegligible issue before its application, especially for those CDs derived from toxic wastes. CCA-treated wood is known to be toxic due to the presence of heavy metals. Thus, assays were conducted to investigate the cytotoxicity of the synthesized C-CDs using *Saccharomyces cerevisiae* as the model strain in the current study. The number of strains that grew on the solid agar medium with 5–75 μL·mL^−1^ concentrations of C-CDs showed similar counts compared to the control sample without the CDs (Figure S6). This observation is consistent with the previous literature, in which the effects of the obtained CDs on the cell viability of HeLa cells were ignorable, revealing the good biocompatibility and low cytotoxicity of CDs [31]. Another report showed that the CDs derived from citrus limetta were nontoxic and possessed bactericidal activity against *E.coli* and *Staphylococcus aureus* [7]. 

In the current study, although Cu ions were present in the synthesized C-CDs, they hardly affected the cell bioactivity of *Saccharomyces cerevisiae*. The reason is that copper ions are bound to CDs without affecting cell growth. A previous study revealed that the effects of carbon dots derived from *Actinidia deliciosa* on the cells were selective. The CDs did not affect normal cells (rather than cancer cells) [14]. All these results agree with the previous conclusion that the inhibition of cell proliferation and cell membrane attachment of the micro-organisms enormously depends on the characteristics of the CDs [9]. 

**Figure 6:**
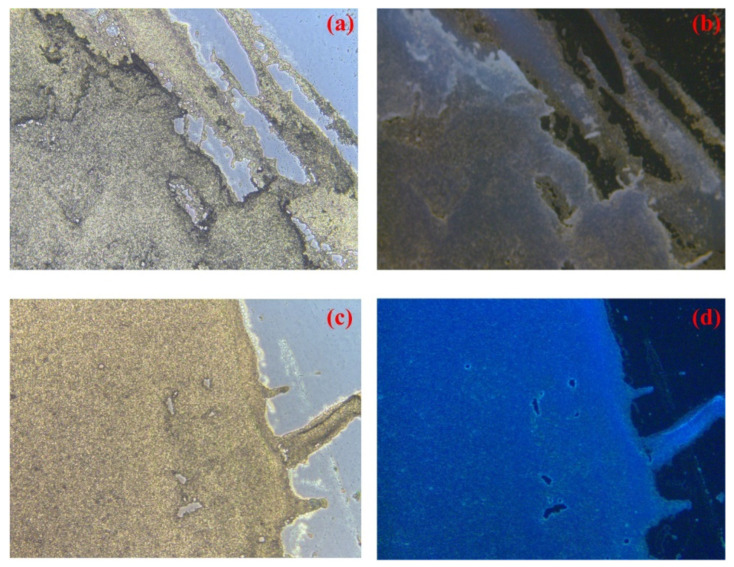
The fluorescence images of *Saccharomyces cerevisiae* under a Leica fluorescence microscope; the cells grown in the medium without the C-CDs in normal vision (**a**) and UV light vision (**b**); the cells grown in the medium with 75 μL·mL^−1^ of C-CDs in normal vision (**c**) and UV light vision (**d**).

Subsequently, the bright blue fluorescence of the strains grown on the CD-doped medium was observed under a Leica fluorescence microscope. The strains in the control medium did not display fluorescence (Figure 6). The results were consistent with a previous study, in which the CDs pass through the insides of the cells and emit fluorescence, showing good bioimaging performance [7]. Copper-doped carbon dots have also been explored for use in oral infectious disease treatment because of their nontoxicity [41]. In summary, the synthesized CDs derived from waste CCA-treated wood have potential use as a great bioimaging reagent and friendly material for biomedical use. 

**Figure 7:**
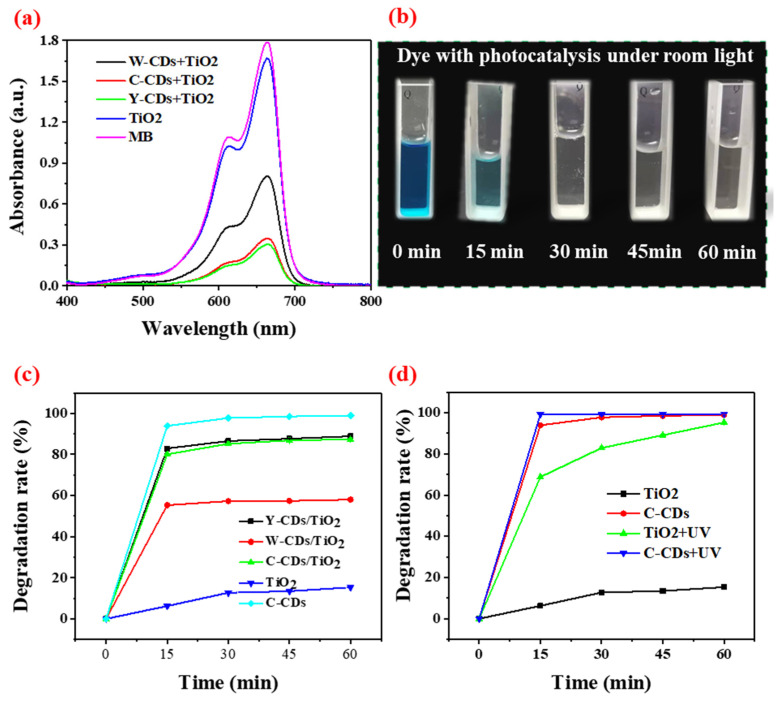
Absorption spectra of the MB solution with a 15 mg·L^−1^ concentration treated by the TiO_2_ and TiO_2_/CDs composites for 15 min under visible light (**a**); representative images of MB degradation by the C-CDs, collected at different times under visible light (**b**); degradation efficiency of MB with different catalysts under room light (**c**), and photocatalytic degradation rate of MB by TiO_2_ and the C-CDs under visible and UV light (**d**).

Photocatalytic degradation is another important application filed for CDs. However, the previous literature reports that pure CDs have no photocatalytic effect and can only be combined with semiconductors [30]. CD/TiO_2_ composites were prepared for the further photocatalysis degradation of methylene blue (MB) dye. The absorption intensity of MB at 664 nm (characteristic peak) was recorded to analyze the dye degradation. Figure 7a shows the UV absorption intensity of the MB dye aqueous solution with the catalysts at different times. The absorption peak intensity of MB decreased with increasing reaction time. The digital image shows that the color of the MB solution gradually becomes lighter with the prolongation of the reaction time (Figure 7b). Those observations are consistent with previous studies [42]. CDs/TiO_2_ catalysts exited higher photocatalytic efficiency for MB dye degradation than the pure TiO_2_ catalysts, especially under visible light (Figure 7c,d and Figure S7a). This finding agrees with previous work, wherein CDs were added to facilitate electron transfer [5,7]. Y-CDs/TiO_2_, C-CDs/TiO_2_, and W-CDs/TiO_2_ photocatalytically degraded the MB solution by 86.5%, 85.2%, and 57.2%, respectively, for 30 min under the visible light from a bulb. An MB removal rate of 12.8% was observed for pure TiO_2_ for the same duration. TiO_2_ has a narrow bandgap and can only absorb a little bit of visible light (<5%). TiO_2_ was commonly doped with other ions for the photocatalytic degradation of dyes [43]. The CDs in the catalyst composites play two main roles in the degradation of MB [8]. Under visible light, the CDs absorb the radiation and then generate electrons and holes. TiO_2_ accepts the generated electrons from the CDs, thus transferring them to the dissolved oxygen in the solution. Under UV irradiation, the CDs transfer the electrons produced by TiO_2_, leading to the charge carrier recombination rate decreasing. Then, the electrons generate reactive oxygen radicals, and the holes contribute to the generation of -OH ions. The previous report also shows copper-doped carbon dots have great photocatalytic efficiency because of the increase in electron transfer rate and charge carrier lifetime [44].

**Scheme 1:**
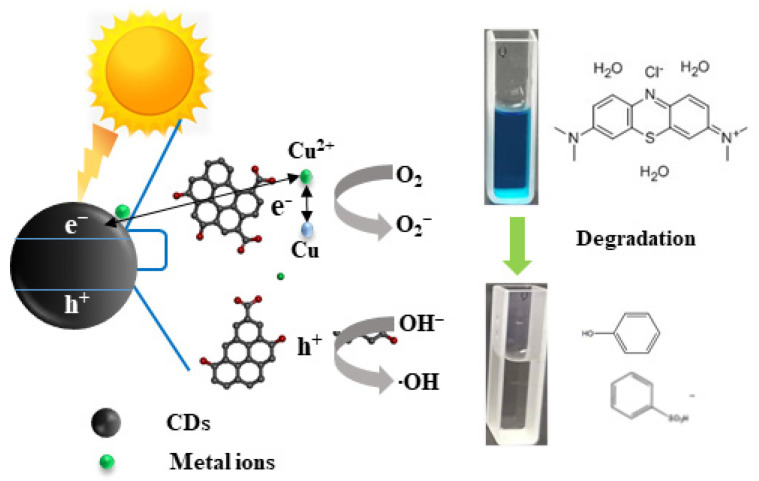
The photocatalytic degradation mechanism of MB by the CDs obtained from the CCA-treated wood waste.

Interestingly, the photocatalytic activity of the copper-doped C-CDs is higher than that of C-CDs/TiO_2_ and pure TiO_2_. The degradation rate of MB was about 97.8% after 30 min using the synthesized C-CDs under visible light. Thus, the synthesized C-CDs remained in nanoparticle states after the degradation of MB. The MB liquid, after photocatalysis, kept its fluorescence characteristics (Figure S7b). The previous literature [11] indicates that nitrogen doping increases the photocatalytic activity of CDs in the reductive photocleavage of N-methyl-4-picolinium esters. Ion doping is an effective way of improving the properties of nanoparticles [45,46]. The porous properties of the CDs and composites were determined by N_2_ adsorption experiments. The N_2_ adsorption test was conducted at 77.3 K using a gas adsorption analyzer (Micromeritics model ASAP-2020, Atlanta, GA, USA). The samples were characterized using the parameters of BET-specific surface area (S_BET_), external surface (S_ext_), total pore volume (V_T_), micropore volume (V_mi_), and mesopore volume (V_me_). Generally, a catalyst with a high specific surface area has great photocatalytic efficiency. In this study, the S_BET_ of C-CDs/TiO_2_ (144.63 m^2^·g^−1^) was greater than that of the C-CDs (80.50 m^2^·g^−1^). However, the C-CDs have greater photocatalytic efficiency than C-CDs/TiO_2_. The reason may be that the C-CDs have a stronger light capture ability than C-CDs/TiO_2_ under visible light. Besides, the presence of Cu ions increased the electron transfer rate of the nano-photoactive catalysts, which is consistent with the previous literature [44]. Those observations indicated that CD properties depend on their compositions and chemical structures. The presence of copper ions as electron acceptors enhanced the catalytic ability of the CDs. The copper ions accepted the photogenerated electrons from the CDs and transferred them to the dissolved oxygen present in the solution, facilitating the photo-oxygen catalysis of MB. The photocatalytic degradation mechanism of the C-CDs towards MB is proposed in Scheme 1. These observations showed that the synthesized C-CDs derived from the photocatalytic properties of the CCA-treated wood were superior to the CDs derived from the original wood and the CDs/TiO_2_ composites. 

The solution proposed here applies to the recycling of all preservative wood waste. Due to the high number of wood products, such as wood utility poles, buildings, and railings, wood waste is a great potential carbon source for CDs. Recycling wood waste can slow carbon emissions and deforestation. The approach proposed here to prepare nanocarbon materials from wood is sustainable. The various applications of CDs have been explored in many studies, as shown in Table 1. However, the CDs prepared in this study possess more outstanding properties, as reflected in the superior photocatalytic activity for organic dye degradation and nontoxicity. The physicochemical characterization suggests the applicability of the given method for the upcycling of CCA-treated wood waste into functional CDs. Hazardous CCA-treated wood waste was thermally conversed into nontoxic luminescent CDs, which can be used in bioimaging. The obtained copper-doped CDs from CCA wood can efficiently photocatalytically degrade sewage. The approach proposed here benefits both wood waste recycling and organic dye degradation in water.

## 4. Conclusions

In this work, novel copper-doped carbon nanodots were fabricated using hazardous CCA-treated wood waste as a carbon-containing precursor material. The synthesis pathway reported here is low-cost, ecofriendly, and organic solvent-free, making it possible for use in industrial manufacturing. The CDs obtained from the original wood, CCA-treated wood waste, and bioremediation CCA wood waste were characterized and compared. The obtained CDs were homogeneous with a similar average size of approximately 1.8–2.8 nm and a well-dispersed population. The XRD and Raman spectra results indicated that the CDs were predominantly amorphous at the macroscale. A blue emission of 440 nm was observed when the obtained C-CDs were under UV light. The cytotoxicity assay demonstrated that the obtained CDs had negligible effects on the growth of *Saccharomyces cerevisiae*, even with a high concentration of C-CDs in the medium. This confirms that they can be used as rare earth-free, ecofriendly, nontoxic, and low-cost phosphors for bioimaging. Heteroatom doping changes the structure and properties of carbon dots. For the Y-CDs, the nitrogen ions prevented the formation of the aromatic ring skeleton and decreased their size. Generally, pure carbon dots do not have a photocatalytic effect. However, due to the presence of copper, a strong photocatalytic effect in the reduction of MB was observed for the obtained C-CDs. Under indoor illumination, the CDs photocatalyzed the degradation of MB; the degradation rate was about 97.8% in 30 min. This observation reveals that the CDs directly obtained from CCA-treated wood waste can be used for photocatalytic wastewater treatment. The reported approach can extend to the reuse of wood waste as a sustainable resource, which provides a great potential way for producing metal-doped CDs via an ecofriendly and wide-ranging preparation method. Functional carbon nanodots have emerged as an urgent material due to their growing use in wide-ranging practical fields.

## Figures and Tables

**Table 1:** Carbon dot synthesis from different precursors and their bio-applications.

Precursors	Synthesized Methods and Conditions	Size (nm)	Applications	Ref.
Quince fruit powder	Hydrothermal; 200 °C, 4 h	4.85 ± 0.07	Cell imaging and Fluorometric detection of As^3+^	[35]
Eleusine coracana	Thermal treatment; 300 °C, 3 h	3–8	Metal ions detection	[32]
Citrus limetta	Pyrolysis; 190 °C, 20 min	4–7	Photoelectrodes and photocatalytic activity	[7]
Mushroom	Hydrothermal; 200 °C, 6 h	2.3 ± 1.3	Hyaluronic acid and hyaluronidase sensing	[47]
Citric acid and thiourea	Microwave treatment;	2–3.5	Bioimaging	[31]
Glucose	hydrothermally treated at 200 °C, 6 h and pyrolysis carbonization at 250 °C, 2 h	1.5–4	Detection of Pb^2+^ ion	[39]
Chitosan	Hydrothermal; 180 °C, 12 h	1.2–2.8	Iodine ions detection	[48]
CCA treated wood	Hydrothermal; 200 °C, 6 h	2–4.5	Bioimaging	This study

## Data Availability

Date will be made available on required.

## Supplementary Materials

The following supporting information can be downloaded at: https://www.mdpi.com/article/10.3390/polym15010136/s1, Figure S1: HR-TEM images of the synthesized CDs; (a,b) W-CDs at 20 and 5 nm scale; (c,d) Y-CDs at 10 and 5 nm scale, respectively; Figure S2: SEM images of CCA-treated wood, (a) before treatment and (b) after treatment using yeasts. Figure S3: High-resolution XP spectrum of O1s of W-CDs, C-CDs, and Y-CDs; Figure S4: (a) Fluorescence spectra of synthesized CDs at different excitation wavelengths from 260 to 300 nm and (b) from 310 to 400 nm for W-CDs; (c) from 260 to 300 nm and (d) from 310 to 400 nm for Y-CDs; Figure S5: digital images of growth characteristics of *Saccharomyces cerevisiae*, (a) the medium without CDs and (b–f) the medium with adding 5, 10, 25, 50, 75 μL/mL of C-CDs, respectively; Figure S6: (a) Degradation efficiency of MB (15 mg·L^−1^) with different catalysts under 365 nm UV light; (b) digital images of the degradation of MB using the synthesized C-CDs at different time intervals under room light or in the 365 nm UV light.

## References

[1] Khan Y., Hwang S., Braveenth R., Jung Y.H., Walker B., Kwon J.H. (2022). Synthesis of fluorescent organic nano-dots and their application as efficient color conversion layers. Nat. Commun..

[2] Khan F.S.A., Mubarak N.M., Tan Y.H., Khalid M., Karri R.R., Walvekar R., Abdullah E.C., Nizamuddin S., Mazari S.A. (2021). A comprehensive review on magnetic carbon nanotubes and carbon nanotube-based buckypaper for removal of heavy metals and dyes. J. Hazard. Mater..

[3] Thaveemas P., Chuenchom L., Kaowphong S., Techasakul S., Saparpakorn P., Dechtrirat D. (2021). Magnetic carbon nanofiber composite adsorbent through green in-situ conversion of bacterial cellulose for highly efficient removal of bisphenol A. Bioresour. Technol..

[4] Kim J., Jang W., Kim J.H., Yang C.-M. (2021). Synthesis of graphene quantum dots-coated hierarchical CuO microspheres composite for use as binder-free anode for lithium-ion batteries. Compos. Part B Eng..

[5] Li H., He X., Kang Z., Huang H., Liu Y., Liu J., Lian S., Tsang C.H.A., Yang X., Lee S.T. (2010). Water-soluble fluorescent carbon quantum dots and photocatalyst design. Angew. Chem. Int. Ed..

[6] Chellasamy G., Arumugasamy S.K., Govindaraju S., Yun K. (2022). Green synthesized carbon quantum dots from maple tree leaves for biosensing of Cesium and electrocatalytic oxidation of glycerol. Chemosphere.

[7] Thakur A., Sharma P.D., Saini S., Jain R., Kumar P. (2019). Citrus limetta Organic Waste Recycled Carbon Nanolights: Photoelectro Catalytic, Sensing, and Biomedical Applications. ACS Sustain. Chem. Eng..

[8] Akbar K., Moretti E., Vomiero A. (2021). Carbon dots for photocatalytic degradation of aqueous pollutants: Recent advancements. Adv. Opt. Mater..

[9] Mahle R., Kumbhakar P., Nayar D., Narayanan T.N., Sadasi V., Uni K.K., Tiwary C.S., Banerjee R. (2021). Current advances in bio-fabricated quantum dots emphasising the study of mechanisms to diversify their catalytic and biomedical applications. Dalton Trans..

[10] Guo J., Lu W., Zhang H., Meng Y., Du F., Shuang S., Dong C. (2021). Copper doped carbon dots as the multi-functional fluorescent sensing platform for tetracyclines and pH. Sens. Actuators B Chem..

[11] Cailotto S., Negrato M., Daniele S., Luque R., Selva M., Amadio E., Perosa A. (2020). Carbon dots as photocatalysts for organic synthesis: Metal-free methylene–oxygen-bond photocleavage. Green Chem..

[12] Liu X., Zhong Q., Guo W., Zhang W., Ya Y., Xia Y. (2021). Novel Platycladus orientalis–shaped Fe-doped ZnO hierarchical nanoflower decorated with Ag nanoparticles for photocatalytic application. J. Alloy. Compd..

[13] Cheng C., Liang Q., Yan M., Liu Z., He Q., Wu T., Luo S., Pan Y., Zhao C., Liu Y. (2022). Advances in preparation, mechanism and applications of graphene quantum dots/semiconductor composite photocatalysts: A review. J. Hazard. Mater..

[14] Arul V., Sethuraman M.G. (2018). Facile green synthesis of fluorescent N-doped carbon dots from Actinidia deliciosa and their catalytic activity and cytotoxicity applications. Opt. Mater..

[15] Guo F., Bao L., Wang H., Larson S.L., Ballard J.H., Knotek-Smith H.M., Zhang Q., Wang X., Han F. (2020). A simple method for the synthesis of biochar nanodots using hydrothermal reactor. MethodsX.

[16] Danial W.H., Abdullah M., Bakar M.A.A., Yunos M.S., Ibrahim A.R., Iqbal A., Adnan N.N. (2022). The valorisation of grass waste for the green synthesis of graphene quantum dots for nonlinear optical applications. Opt. Mater..

[17] Ma Z., Ming H., Huang H., Liu Y., Kang Z. (2012). One-step ultrasonic synthesis of fluorescent N-doped carbon dots from glucose and their visible-light sensitive photocatalytic ability. New J. Chem..

[18] Prasannan A., Imae T. (2013). One-Pot Synthesis of Fluorescent Carbon Dots from Orange Waste Peels. Ind. Eng. Chem. Res..

[19] Shi C., Qi H., Ma R., Sun Z., Xiao L., Wei G., Huang Z., Liu S., Li J., Dong M. (2019). N,S-self-doped carbon quantum dots from fungus fibers for sensing tetracyclines and for bioimaging cancer cells. Mater. Sci. Eng. C.

[20] Tan X.W., Romainor A., Chin S.F., Ng S.M. (2014). Carbon dots production via pyrolysis of sago waste as potential probe for metal ions sensing. J. Anal. Appl. Pyrolysis.

[21] Zhang Z., Liu J., Shen F., Dong Y. (2020). Temporal influence of reaction atmosphere and chlorine on arsenic release in combustion, gasification and pyrolysis of sawdust. J. Hazard. Mater..

[22] Perez J.P.H., Schiefler A.A., Rubio S.N., Reischer M., Overheu N.D., Benning L.G., Tobler D.J. (2021). Arsenic removal from natural groundwater using ‘green rust’: Solid phase stability and contaminant fate. J. Hazard. Mater..

[23] Maietta M., Colombo R., Lavecchia R., Sorrenti M., Zuorro A., Papetti A. (2017). Artichoke (*Cynara cardunculus* L. var. scolymus) waste as a natural source of carbonyl trapping and antiglycative agents. Food Res. Int..

[24] Kim J.-Y., Oh S., Park Y.-K. (2020). Overview of biochar production from preservative-treated wood with detailed analysis of biochar characteristics, heavy metals behaviors, and their ecotoxicity. J. Hazard. Mater..

[25] Braghiroli F.L., Cuña A., da Silva E.L., Amaral-Labat G., e Silva G.F.B.L., Bouafif H., Koubaa A. (2020). The conversion of wood residues, using pilot-scale technologies, into porous activated biochars for supercapacitors. J. Porous Mater..

[26] Nagamalai V., Vania V.B., Juan G., Fátima C., Mario M.M., Manuel C., Lorena D., Begoña E., Teresa F. (2018). Green synthesis of fluorescent carbon dots from spices for in vitro imaging and tumour cell growth inhibition. Beilstein J. Nanotechnol..

[27] Zhou Z., Tian P., Liu X., Mei S., Ding Z., Di L., Jing P., Zhang W., Guo R., Qu S. (2018). Hydrogen Peroxide-Treated Carbon Dot Phosphor with a Bathochromic-Shifted, Aggregation-Enhanced Emission for Light-Emitting Devices and Visible Light Communication. Adv. Sci..

[28] Martindale B., Hutton G., Caputo C.A., Prantl S., Godin R., Durrant J.R., Reisner E. (2017). Enhancing Light Absorption and Charge Transfer Efficiency in Carbon Dots Through Graphitization and Core Nitrogen Doping. Angew. Chem..

[29] Ding H., Li X.-H., Chen X.-B., Wei J.-S., Li X.-B., Xiong H.-M. (2020). Surface states of carbon dots and their influences on luminescence. J. Appl. Phys..

[30] Kou X., Xin X., Zhang Y., Meng L.Y. (2021). Facile synthesis of nitrogen-doped carbon dots (N-CDs) and N-CDs/NiO composite as an efficient electrocatalyst for oxygen evolution reaction. Carbon Lett..

[31] Li H., Shao F.-Q., Huang H., Feng J.-J., Wang A.-J. (2016). Eco-friendly and rapid microwave synthesis of green fluorescent graphitic carbon nitride quantum dots for vitro bioimaging. Sens. Actuators B Chem..

[32] Murugan N., Prakash M., Jayakumar M., Sundaramurthy A., Sundramoorthy A.K. (2019). Green synthesis of fluorescent carbon quantum dots from Eleusine coracana and their application as a fluorescence ‘turn-off’ sensor probe for selective detection of Cu^2+^. Appl. Surf. Sci..

[33] Lin Z., Song X., Xu Y. (2017). Study on the Infrared Spectral Characteristic of *Tetracentron sinense* Wood. Hubei Agric. Sci..

[34] Liu Y., Guo D., Gao Y., Tong B., Li Y., Zhu Y. (2021). Non-thermal effect of microwave on the chemical structure and luminescence properties of biomass-derived carbon dots via hydrothermal method. Appl. Surf. Sci..

[35] Ramezani Z., Qorbanpour M., Rahbar N. (2018). Green synthesis of carbon quantum dots using quince fruit (*Cydonia oblonga*) powder as carbon precursor: Application in cell imaging and As3+ determination. Colloids Surf. Physicochem. Eng. Asp..

[36] Kaczmarek A., Hoffman J., Morgiel J., Mościcki T., Stobiński L., Szymański Z., Małolepszy A. (2021). Luminescent Carbon Dots Synthesized by the Laser Ablation of Graphite in Polyethylenimine and Ethylenediamine. Materials.

[37] Mikailzade F., Türkan H., Önal F., Karataş Ö., Kazan S., Zarbali M., Göktaş A., Tumbul A. (2020). Structural, optical and magnetic characterization of nanorod-shaped polycrystalline Zn1− xMnxO films synthesized using sol–gel technique. Appl. Phys. A.

[38] Goktas A., Modanlı S., Tumbul A., Kilic A. (2022). Facile synthesis and characterization of ZnO, ZnO:Co, and ZnO/ZnO:Co nano rod-like homojunction thin films: Role of crystallite/grain size and microstrain in photocatalytic performance. J. Alloy. Compd..

[39] Jing S., Zhao Y., Sun R.-C., Zhong L., Peng X. (2019). Facile and High-Yield Synthesis of Carbon Quantum Dots from Biomass-Derived Carbons at Mild Condition. ACS Sustain. Chem. Eng..

[40] Feng T., Ai X., An G., Yang P., Zhao Y. (2016). Charge-Convertible Carbon Dots for Imaging-Guided Drug Delivery with Enhanced in Vivo Cancer Therapeutic Efficiency. ACS Nano.

[41] Liu M., Huang L., Xu X., Wei X., Yang X., Li X., Wang B., Xu Y., Li L., Yang Z. (2022). Copper Doped Carbon Dots for Addressing Bacterial Biofilm Formation, Wound Infection, and Tooth Staining. ACS Nano.

[42] Daneshvar E., Vazirzadeh A., Niazi A., Kousha M., Naushad M., Bhatnagar A. (2017). Desorption of Methylene blue dye from brown macroalga: Effects of operating parameters, isotherm study and kinetic modeling. J. Clean. Prod..

[43] Zuorro A., Lavecchia R., Monaco M.M., Iervolino G., Vaiano V. (2019). Photocatalytic Degradation of Azo Dye Reactive Violet 5 on Fe-Doped Titania Catalysts under Visible Light Irradiation. Catalysts.

[44] Xue N., Kong X., Song B., Bai L., Zhao Y., Lu C., Shi W. (2017). Cu-Doped Carbon Dots with Highly Ordered Alignment in Anisotropic Nano-Space for Improving the Photocatalytic Performance. Sol. RRL.

[45] Montanaro D., Lavecchia R., Petrucci E., Zuorro A. (2017). UV-assisted electrochemical degradation of coumarin on boron-doped diamond electrodes. Chem. Eng. J..

[46] Goktas A. (2020). Role of simultaneous substitution of Cu2+ and Mn2+ in ZnS thin films: Defects-induced enhanced room temperature ferromagnetism and photoluminescence. Phys. E Low-Dimens. Syst. Nanostruct..

[47] Yang K., Liu M., Wang Y., Wang S., Miao H., Yang L., Yang X. (2017). Carbon dots derived from fungus for sensing hyaluronic acid and hyaluronidase. Sens. Actuators B Chem..

[48] Song J., Zhao L., Wang Y., Xue Y., Deng Y. (2018). Carbon Quantum Dots Prepared with Chitosan for Synthesis of CQDs/AuNPs for Iodine Ions Detection. Nanomaterials.

