# Supplementary material for: Copper-Doped Carbon Nanodots with Superior Photocatalysis, Directly Obtained from Chromium-Copper-Arsenic-Treated Wood Waste
Source: Polymers (Basel). 2022 Dec 28;15(1):136. doi: 10.3390/polym15010136 (PMC9823717; doi:10.3390/polym15010136)
Supplement: Supplementary file 1 [file polymers-15-00136-s001.zip › polymers-2087979-supplementary.pdf]

Copper-Doped Carbon Nanodots with Superior Photocatalysis, Directly Obtained from Chromium-Copper-Arsenic-Treated Wood Waste

Dan Xing 1,2, Ahmed Koubaa 1,*, Yubo Tao 2, Sara Magdouli 3, Peng Li 2, Hassine Bouafif 3 and Jingfa Zhang 1,2,*

1 Forest Research Institute, Université du Québec en Abitibi-Témiscamingue, Rouyn-Noranda, QC J9X 5E4, Canada
2 State Key Laboratory of Biobased Material and Green Papermaking, Qilu University of Technology, Shandong Academy of Sciences, Jinan 250353, China
3 Centre Technologique des Résidus Industriels (CTRI), 433 Boulevard du Collège, Rouyn-Noranda, QC J9X 0E1, Canada
* Correspondence: ahmed.koubaa@uqat.ca (A.K.); jfzhang@qlu.edu.cn (J.Z.)

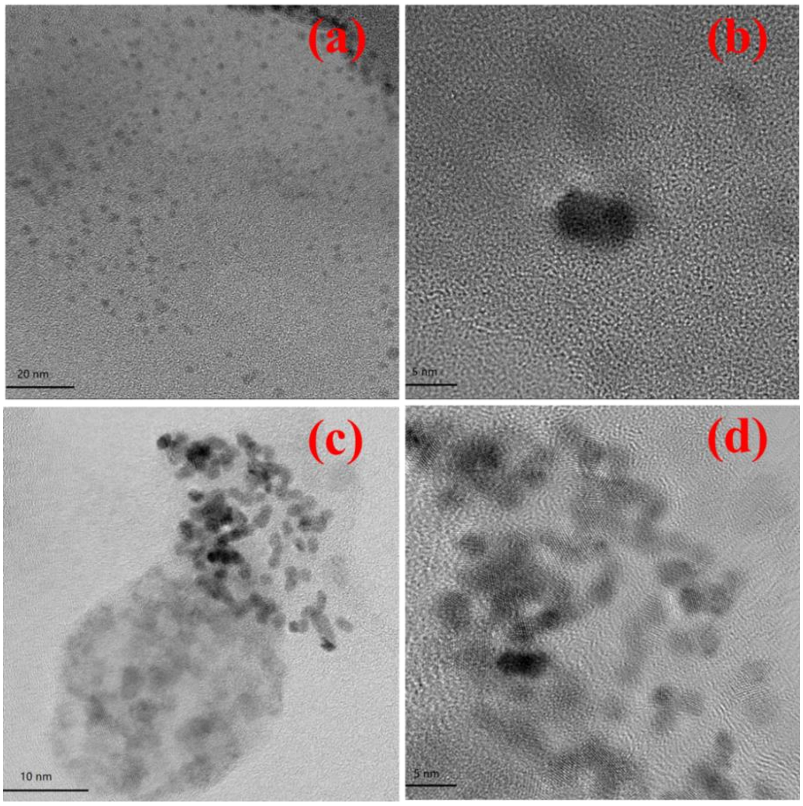

Fig. S1 HR-TEM images of the synthesized CDs; (a) and (b) W-CDs at 20 and 5 nm scale; (c) and (d) Y-CDs at 10 and 5 nm scale, respectively.

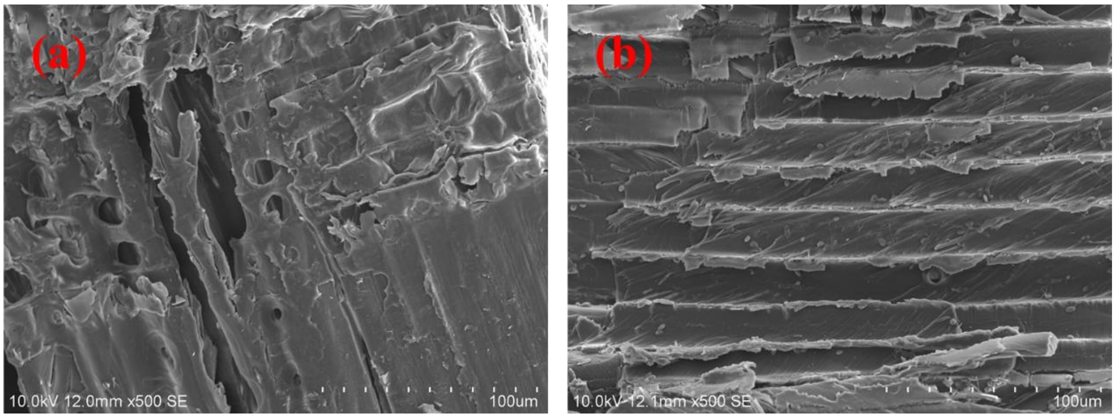

Fig. S2 SEM images of CCA-treated wood, (a) before treatment and (b) after treatment using yeasts.

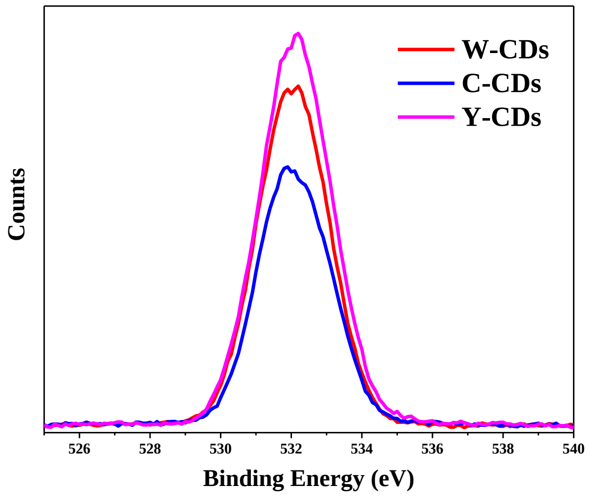

Fig. S3 High resolution XP spectrum of O1s of W-CDs, C-CDs, and Y-CDs.

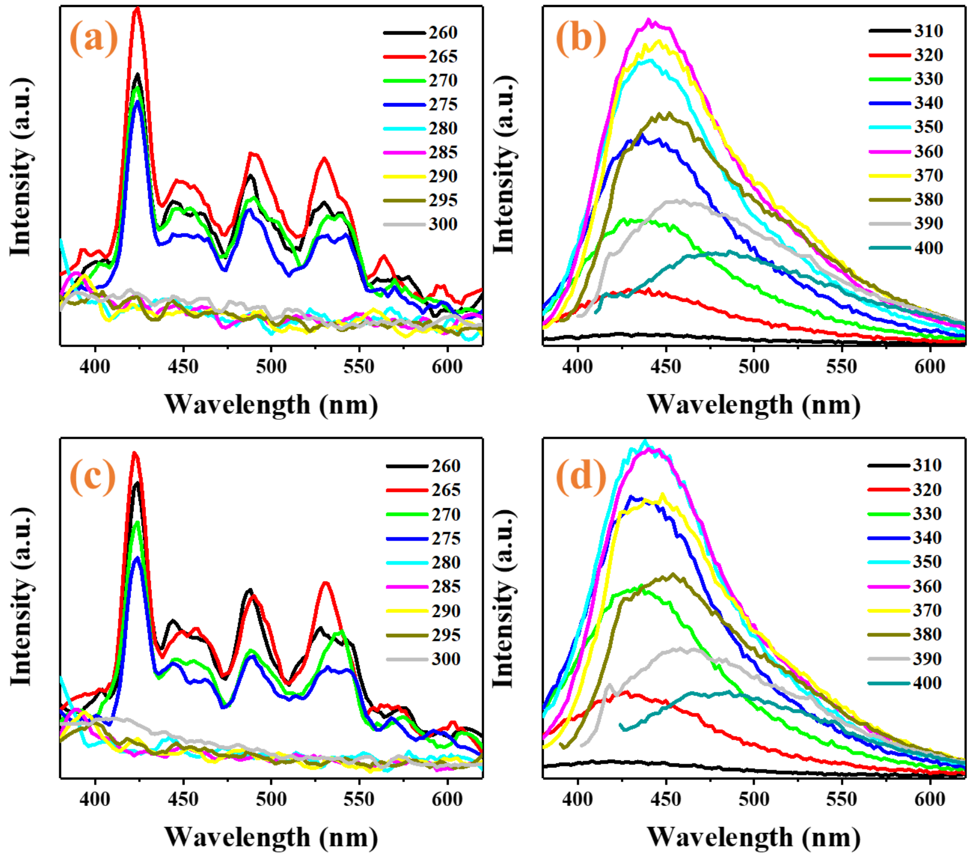

Fig. S4 (a) Fluorescence spectra of synthesized CDs at different excitation wavelengths from 260 to 300 nm and (b) from 310 to 400 nm for W-CDs; (c) from 260 to 300 nm and (d) from 310 to 400 nm for Y-CDs.

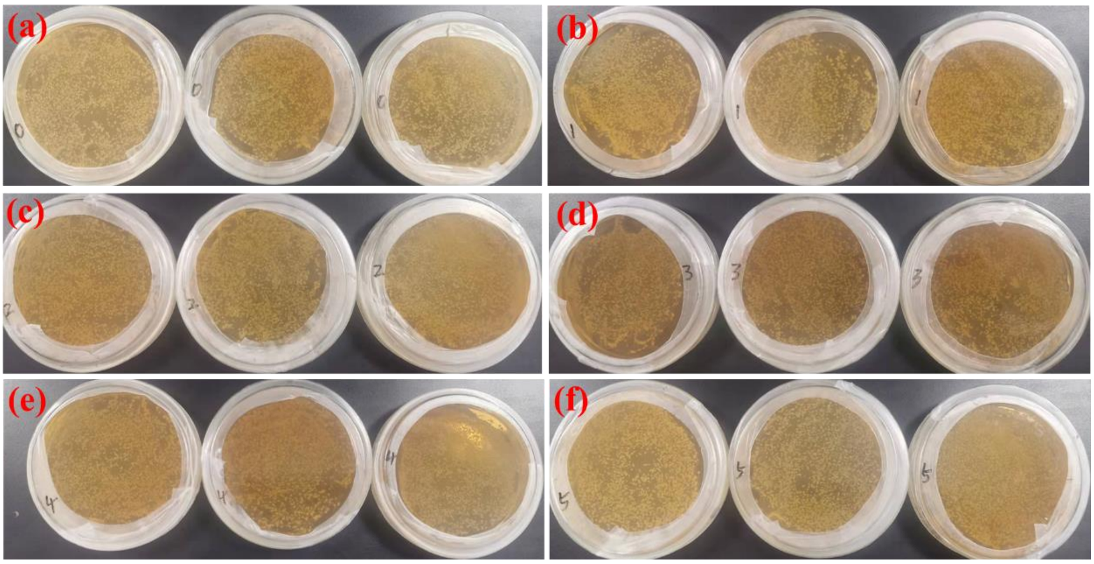

Fig. S5 digital images of growth characteristics of *Saccharomyces cerevisiae*, (a) the medium without CDs and (b)-(f) the medium with adding 5, 10, 25, 50, 75 μL/mL of C-CDs, respectively.

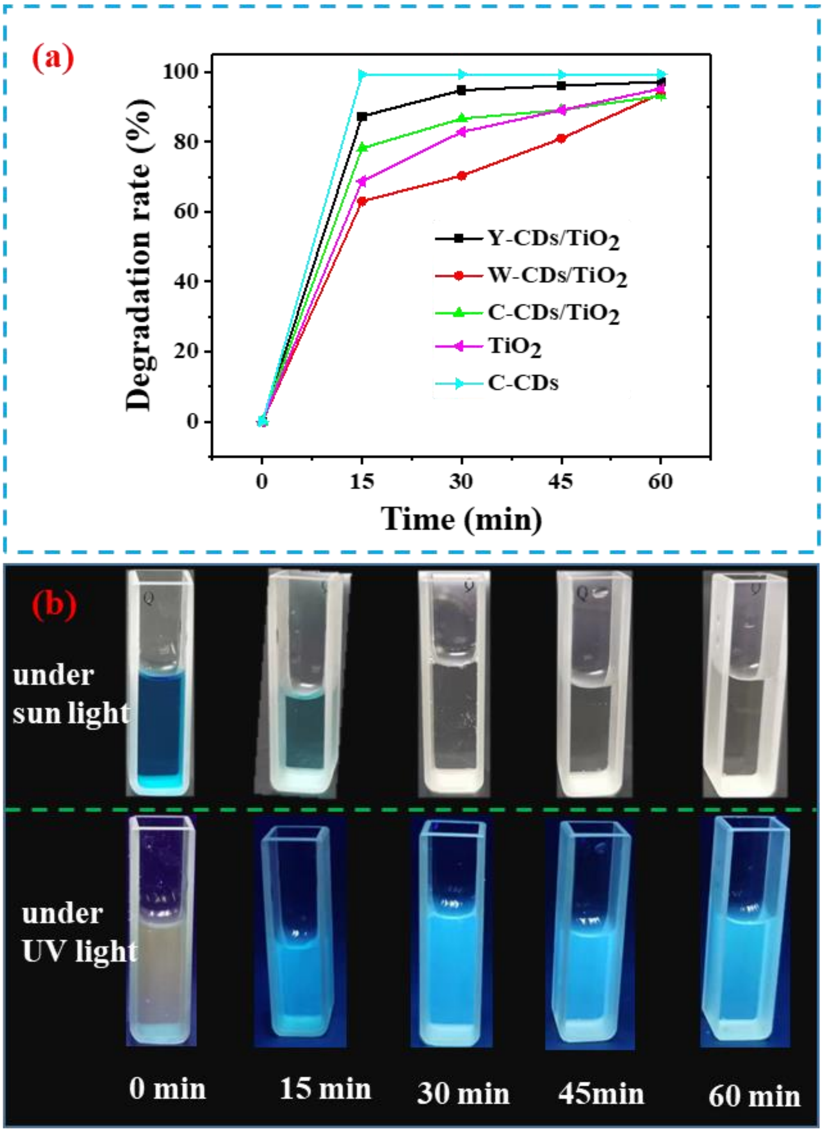

Fig. S6 (a) Degradation efficiency of MB (15 mg·L^{-1}) with different catalysts under 365 nm UV light; (b) digital images of the degradation of MB using the synthesized C-CDs at different time intervals under room light or in the 365 nm UV light.
